# Evaluation of upper extremity ataxia through image processing in individuals with multiple sclerosis

**DOI:** 10.1590/0004-282X-ANP-2020-0587

**Published:** 2021-12-17

**Authors:** Fatma Erdeo, İbrahim Yildiz, Ali Ulvi Uca, Mustafa Altaş

**Affiliations:** 1 Necmettin Erbakan University, Faculty of Health Science, Konya, Turkey.; 2 Necmettin Erbakan University, Faculty of Engineering, Konya, Turkey.; 3 Necmettin Erbakan University, Faculty of Medicine, Konya, Turkey.

**Keywords:** Multiple Sclerosis, Ataxia, Upper Extremity, Diagnosis, Esclerose Múltipla, Ataxia, Extremidade Superior, Diagnóstico

## Abstract

**Background:**

Impaired dexterity is a frequently reported disability among people with ataxic multiple sclerosis (MS).

**Objective:**

To quantify and standardize the evaluation of upper extremity coordination disorder among patients with multiple sclerosis (MS), using the Tablet Ataxia Assessment Program (TAAP).

**Methods:**

The X and Y axis movements of 50 MS patients and 30 healthy individuals who were evaluated using the International Cooperative Ataxia Rating Scale (ICARS) were also assessed using TAAP. The functional times of the participants’ right and left hands were recorded using the nine-hole peg test (NHPT). The upper extremity coordination of individuals with MS was evaluated using the upper extremity kinetic functions section of ICARS.

**Results:**

The deviations for the X and Y axis movements of the MS group were greater than those of the control group (p<0.05). Significant correlations were shown between TAAP scores and NHPT and ICARS scores. The strongest correlation was found between NHPT and ICARS in the dominant hand (r_nhpt_=0.356, p_nhpt_=0.001; r_icars_=0.439, p_icars_=0.000). In correlating the Y axis with ICARS, the deviations in the Y axis were found to be greater in the non-dominant hand than those in the X axis (r_yright_=0.402, p_yright_=0.004; r_yleft_=0.691, p_yleft_=0.000).

**Conclusion:**

Measurement using TAAP is more sensitive than other classical and current methods for evaluating ataxia. We think that TAAP is an objective tool that will allow neurorehabilitation professionals and clinicians to evaluate upper extremity coordination.

## RESUMO

**Antecedentes:**

Destreza prejudicada é uma deficiência frequentemente relatada em pessoas com esclerose múltipla (EM) atáxica.

**Objetivo:**

Nosso objetivo é quantificar e padronizar a avaliação do distúrbio de coordenação da extremidade superior em pacientes com EM usando o *Tablet Ataxia Assessment Program* (TAAP).

**Métodos:**

Os movimentos dos eixos X e Y de 50 EM e 30 indivíduos saudáveis que foram avaliados com a *International Cooperative Ataxia Rating Scale* (ICARS) também foram avaliados com TAAP. Os tempos funcionais das mãos direita e esquerda dos participantes foram registrados usando o teste de nove pinos (NHPT). A coordenação da extremidade superior de indivíduos com EM foi avaliada com a seção de funções cinéticas da extremidade superior do ICARS.

**Resultados:**

Os desvios para os movimentos dos eixos X e Y do grupo MS foram maiores do que os do grupo controle (p <0,05). Correlações significativas foram mostradas entre os escores do TAAP e os escores do NHPT e do ICARS. A correlação mais forte foi encontrada entre NHPT e ICARS na mão dominante (r_nhpt_=0,356, p_nhpt_=0,001, r_icars_=0,439 e p_icars_=0,000). Na correlação do eixo Y com ICARS, observou-se que os desvios no eixo Y foram maiores na mão não dominante do que no eixo X (r_yright_=0,402, p_yright_=0,004, r_yleft=_0,691 e p_yleft_=0,000).

**Conclusão:**

A medição com TAAP é mais sensível do que outros métodos clássicos e atuais de avaliação de ataxia. Acreditamos que o TAAP seja uma ferramenta objetiva que permitirá aos profissionais de neurorreabilitação e médicos avaliar a coordenação dos membros superiores.

## INTRODUCTION

Ataxia is defined as a disorder in the coordination of voluntary muscle movement. It is not a disease but, rather, a physical finding^1^ and is seen in approximately 75% of patients with multiple sclerosis (MS)^2^. Ataxia can present as trunk or limb ataxia or as a combination of the two. While trunk ataxia results from midline damage in the cerebellar vermis and associated pathways, extremity ataxia can be lateralized by ipsilateral cerebellar lesions^1^. Lower extremity ataxia is defined as gait disturbances. Upper extremity ataxia is characterized by tremor and dysynergy^3^. In upper extremity ataxia, disturbances are observed in daily life activities such as inability to write, inability to fasten buttons and difficulty in picking up small objects. Extremity ataxia is clinically evaluated by means of the toe-nose and knee-heel test^3^.

In addition to surgical and pharmacological treatments, physiotherapy modalities such as exercise, thermal applications and electrotherapy are widely used to cope with ataxic symptoms. Various scales such as the International Cooperative Ataxia Rating Scale (ICARS)^4^ and the Ataxia Assessment and Rating Scale (SARA)^5^ are used to detect ataxia in MS patients and to verify the effectiveness of treatments. These assessment methods consist of sub-parameters evaluating posture, gait, speech and upper extremity performance. On these scales, which are based on subjective evaluations by observers, terms such as “no sensitivity,” “mild,” “moderate” and “severe” are used in assessing disability.

However, new assessment methods that are one level higher than classical evaluations are also used in assessing ataxia. Two of these are the nine-hole peg test (NHPT) and the box block test (BBT). NHPT is part of the Multiple Sclerosis Functional Composite (MSFC). For MS clinical studies, the MSFC measures an outcome. MSFC correlates better with magnetic resonance imaging (MRI) variables than does EDSS^6,7^. It also correlates significantly with the disease-related quality of life reported by the patient^8^.

NHPT has advantages, as well as mild drawbacks. It has been reported in studies that this test is not sensitive enough to detect mild impairment in manual dexterity (EDSS<3) in individuals, and that NHPT scores vary greatly in severely disabled individuals (EDSS>6.0)^9^. In addition, material is required for NHPT and BBT tests, and the inability to provide a comfortable evaluation create disadvantages for clinicians. It is obvious that there is a need for more sensitive, easier-to-apply and more reproducible tests for evaluating upper extremity function.

While data can often be obtained easily and quickly through the development of technology, the inadequacy of ataxia clinical rating systems such as ICARS and SARA, which are still in use, is alarming. Use of valid and reliable assessment tools is extremely important for testing new therapeutic approaches and for setting goals. Therefore, the aim of our observational, cross-sectional study was to evaluate upper extremity ataxia in MS patients using TAAP, which is an objective assessment method. TAAP is the abbreviation for tablet ataxia assessment program. Upper extremity ataxia was investigated using a sensor mounted on the patient’s index finger, and kinematic information was evaluated through a program loaded on the tablet.

## METHODS

### Ethics committee

Written informed consent was obtained from all patients. The study was conducted in accordance with the Declaration of Helsinki and later amendments. Approval was obtained from Necmettin Erbakan University Non-Invasive Clinical Research Ethics Committee.

### Participants

**Figure 1:**
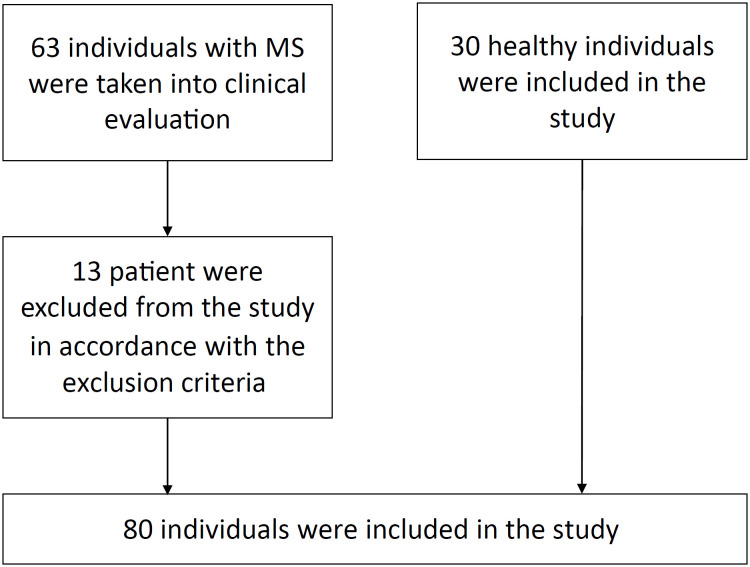
Tablet Ataxia Assessment Protocol.

This study was conducted between December 1, 2019 and October 15, 2020, at the Neurology Clinic of Meram Faculty of Medicine, Necmettin Erbakan University. Sixty-three MS patients whose diagnoses had been made in accordance with the McDonald criteria and 30 healthy individuals were evaluated^10^. Thirteen patients were excluded from the study in accordance with the following exclusion criteria (Figure 1): having an acute attack with impairments in activities such as walking, speaking and vision, in the last three months; presence of orthopedic, neurological (sensory impairment and apraxia) or systemic problems that prevented participation in the evaluations; peripheral vestibular problems; advanced cognitive dysfunction; and increased tonus that affected upper extremity function. Extremity tremor was evaluated by means of spiral drawing from the upper extremity kinetic functions section of ICARS. Patients who scored ≤ 1 in this section were included in the study.

#### Ataxic multiple sclerosis *group*

In accordance with a cerebellar evaluation using the Expanded Disability Status Scale (EDSS), patients with extremity ataxia symptoms alone were included in this group. To eliminate trunk ataxia as much as possible, the following EDSS measurements were applied:

- Trunk ataxia≤1
- Romberg test≤1
- Upper limb ataxia≥1
- Functional reach test≤25 cm

#### Control group

##### Inclusion criteria for healthy individuals

No neurological disease was diagnosed, no dizziness or loss of sensation in the foot and no scars or foot or ankle problems that would affect plantar sensation. The individuals were informed about the purpose and methods of the study.

### Evaluation protocol

#### Clinical evaluation

##### Physical characteristics and history

The patients’ age, height, weight, family history of MS, medications used, last date of corticosteroid use, previous attacks and presence of systemic or orthopedic diseases were recorded in detail.

##### Nine-Hole Peg Test

In Kellor et al., the NHPT as defined by Godkin was applied to MS patients^11^. It has been shown that NHPT for upper extremity rehabilitation assessment and treatment of MS is by far the most commonly used measurement and has been used in 63% of published studies^12^. For this reason, NHPT is widely considered to be a gold standard measurement for dexterity. In this test, firstly, the dominant hand is used to insert nine rods of 3.2 cm in length into the holes in the apparatus, one by one, as quickly as possible. In the second stage, the rods are removed sequentially; the length of time that the patient takes to insert and remove the rods is recorded. The same process is repeated for the other hand^13^.

##### International Ataxia Rating Scale

ICARS is a test developed to evaluate the severity of ataxia^4,14^. The validity and reliability of the Turkish version were established by Salcı et al^15^. In this test, scores for kinetic functions were determined separately for each hand using the finger-nose test (which tests the intentional tremor of the fingers) and the finger-finger test (which tests pronation-supination alternating movements). “Drawing the curved spiral” was omitted from the scoring because it evaluated the dominant hand. Other evaluations of upper extremity kinetic functions were included in the scoring.

##### Expanded Disability Status Scale

EDSS was developed to follow the disease progression of MS through evaluating the brainstem, pyramidal system, cerebellar and cerebral system, vision, sensory problems, bladder-bowel problems and ambulation. The scores obtained from all these functional system (FS) evaluations are converted into a single score, and the severity of the disease is graded between 0 and 10^16^.

### Data analysis

The data were analyzed using software in the Android operating system, through the Opencv library. The software works with the logic of following a 6 mm radius colored point (marker) in images obtained in real time. We determined the color of this point as pink. Through knowing the point diameter, the image can be scaled and sized; therefore, it can be calculated how many mm of displacement the point makes on the vertical and horizontal axes during its movement on the screen.

**Figure 2:**
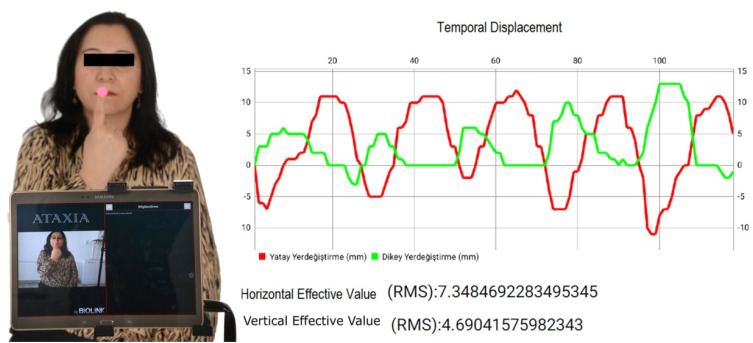
Tablet Ataxia Assessment Program.Table 1. Distribution of descriptive characteristics of multiple sclerosis patients and healthy individuals.

After the software has been started and the images start to flow to the screen, the user selects the “marker” with his finger on the touch screen and, thus, introduces the “marker” color to the software. After this step, the software calculates the displacements of the “marker” in the horizontal and vertical axes (Figure 2). The position of the “marker” at the time at which the “marker” color is defined is accepted as the zero position by the software. This makes error analysis difficult, as the sign of tremors or displacements may also be negative. While calculating the average error amount, the same magnitude of negative error zeroes the positive error. This situation causes the error average of vibration movements of equal amplitude to be obtained as zero.

To avoid this situation, instead of calculating the average error value on the horizontal and vertical axes, the root mean square error (RMSe), which is frequently used in statistics, was used^17^. In the following equation, the RMSe expresses the squared error; the equation shows how the squared error is calculated for a test series consisting of “n” samples.


$$RMSe=\sqrt{\frac{e_{1}^{2}+e_{2}^{2}+\cdots +e_{n}^{2}}{n}}=\sqrt{\frac{\sum_{i=1}^{n}e_{1}^{2}}{n}}$$

### Statistics

IBM’s Statistical Package for the Social Sciences, version 20, analysis program (SPSS Inc., an IBM Company, Chicago, IL, USA) was used for the statistical analysis. Descriptive statistics were used for demographic data. Means±standard deviations and frequency values were used for the measured variables and percentages.

The G*Power software package (G*Power Ver. 3.0.10, Franz Faul, Kiel University, Germany) was used to determine the sample size required for the study^18^. To determine the number of patients in the group, Germatoni et al. was used as a reference^19^. Fifty-one individuals were included in each group, with 80% sample size power (d=0.50 effect width, α=0.05 type I error, β=0.20 type II error).

Normal data distribution was evaluated using the Shapiro-Wilk test. The significance level of our data, which did not have a normal distribution, was analyzed using the Mann-Whitney U test. The relationship between independent variables was examined by means of Spearman correlation analysis. The significance level was taken to be p<0.05 for nonparametric evaluations^20,21^.

## RESULTS

The descriptive characteristics of the participants in the study are provided in Table 1. Fifty MS patients (aged 34.58±8.50 years) and 30 control group individuals (aged 34.66±11.93 years) were included in the study. The EDSS average score was X: 3.18, SD±1.7 (Table 1). The differences in average age and gender of the healthy and MS individuals were evaluated in independent groups by comparing them using a Mann-Whitney U analysis. It was determined that there was no significant difference in average age distributions between the groups (p>0.05; Table 1), and that the groups were homogeneous/similar.

**Table 1:** Distribution of descriptive characteristics of multiple sclerosis patients and healthy individuals.

Features		Multiple sclerosis (n=24)		Control group (n=36)		p-value
Age (years)		34.58±8.52		35.23±12.40		0.944
EDSS		3.18±1.70		–		–
Sex	Male	n=10	20%	n=12	40%	0.054
	Female	n=40	80%	n=18	60%	

N: number; EDSS: Expanded Disability Status Scale; Mann-Whitney U test; p<0.05.

The right hand was dominant in all patients and in the control group. Comparison of the mean NHPT, TAAP and ICARS scores of the right and left hands of the healthy individuals and MS patients with extremity ataxia showed that the differences between the groups were significant (p<0.05; Table 2).

**Table 2:** Comparison of evaluation parameters of multiple sclerosis patients and healthy individuals.

Tests	Multiple sclerosis (n=50)	Control group (n=30)	p-value*
NHPT (right)	24.54±4.34	20.03±1.73	0.000
NHPT (left)	25.96±4.26	21.93±2.25	0.000
X (right)	6.74±3.52	3.93±1.11	0.000
Y (right)	7. 24±2.27	3.07±1.38	0.000
X (left)	8.10±3.81	5.33±1.53	0.000
Y (left)	8.39±2.78	3.43±1.38	0.000
ICARS (right)	5.86±2.33	–	–
ICARS (left)	6.20±2.23	–	–

NHPT: Nine-Hole Peg Test; X: X axis; Y: Y axis; ICARS: International Cooperative Ataxia Scale; data are expressed as mean±SD

* Spearman and Pearson correlation analyses; p<0.05.

### Tablet Ataxia Assessment Program

The ataxia parameters of TAAP on the X and Y axes were significantly different between the groups (p<0.05). In comparisons between groups, the dominant and non-dominant upper extremity ataxia data of the individuals with MS were found to be higher on the X and Y axes than those of the control group. Among individuals with MS, the data on the Y axis of the non-dominant hand were higher than those of the dominant hand (p<0.05) (Table 2).

### Nine-Hole Peg Test

The NHPT has long been regarded as an indicator of hand awkwardness. Comparison between the two groups showed that the NHPT scores for the dominant and non-dominant hand of individuals with MS were higher than those of the control group. Among the individuals with MS, the scores for the non-dominant hand were higher than those for the dominant hand (p<0.05) (Table 2).

The X and Y values of the dominant hand showed a moderate positive correlation with ICARS (r=0.439, p<0.001; r=0.402, p=0.004, respectively). While the TAAP data of the dominant hand shows weak-moderate positive correlation with the data on the X axis, a strong positive correlation with the data on the Y axis was determined (r=0.356 p=0.001; r=0.639, p<0.001, respectively). The X and Y TAAP data of the dominant hand showed weak-moderate positive correlations with EDSS (r=0.353 p=0.012; r=0.334, p=0.018, respectively). In the ICARS kinetic functions section, a strong positive correlation was found between the right and left hand and EDSS (r=0.600, p<0.001; r=0.638, p<0.001, respectively) (Table 3).

**Table 3:** Correlation of evaluation parameters among multiple sclerosis patients and healthy individuals.

		NHPT (right)	NHPT (left)	X (right)	Y (right)	X (left)	Y (left)	ICARS (right)	ICARS (left)	EDSS	Age
NHPT (right)	r p	1.000	0.630** 0.000	0.356** 0.001	0.639** 0.000	0.228* 0.042	0.520** 0.000	0.511** 0.000	0.446** 0.001	0.532* 0.000	0.223* 0.047
NHPT (left)	r p		1.000	0.322* 0.004	0.491** 0.000	0.523** 0.000	0.666** 0.000	0.356* 0.011	0.535** 0.000	0.343** 0.015	0.147 0.308
X (right)	r p			1.000	0.401** 0.000	0.351** 0.001	0.399** 0.000	0.439** 0.000	0.254 0.075	0.353* 0.012	0.177 0.116
Y (right)	r p				1.000	0.236 0.098	0.069 0.634	0.402** 0.004	0.196 0.173	0.334** 0.018	0.080 0.478
X (left)	r p					1.000	0.503** 0.000	0.239 0.095	0.292* 0.040	0.517** 0.000	0.022 0.847
Y (left)	r p						1.000	0.238 0.095	0.691** 0.000	0.292* 0.040	0.182 0.105
ICARS (right)	r p							1.000	0.769** 0.000	0.600** 0.000	0.418** 0.003
ICARS (left)	r p								1.000	0.638** 0.000	0.350* 0.013
EDSS	r p									1.000	0.471** 0.001
Age	r p										1.000

NHPT: Nine-Hole Peg Test; X: X axis; Y: Y axis; ICARS: International Cooperative Ataxia Scale; EDSS: Expanded Disability Status Scale

* difference between groups (p<0.05)

** high level of significance.

## DISCUSSION

In this study, the usability of the TAAP was examined through correlating the X horizontal and Y vertical data obtained from patients with ataxic MS, in order to quantify and standardize functional changes in upper extremity movements, using TAAP (Table 3). Our study differed from studies in which extremity ataxia was evaluated using current methods^22,23^, given that we excluded trunk ataxia as much as possible. It is difficult to evaluate pure limb ataxia. For this reason, some positioning is used to evaluate the extremity ataxia. For example, the knee-heel test is performed in a lying-down position, with the aim of excluding trunk ataxia in the lower extremity.

However, we did not have an alternative for excluding trunk ataxia from assessment of upper extremity ataxia, given that this is performed in a sitting position. Therefore, in the evaluation of upper extremity ataxia, we set some parameters for elimination of trunk ataxia, and we made the evaluation accordingly. The most important result obtained in our study was the fact that the measurement parameters for MS patients with coordination problems in the X and Y axes were higher than those of the control group, and the correlation of TAAP with NHPT was significant. In other words, MS patients with coordination problems showed more deviation from the axis of movement during movement than did the control group.

The methods used to assess cerebellar ataxia in most previous studies were semi-quantitative scales based on subjective predictions by observers (e.g. ICARS^4^, SARA^5^ and Short Ataxia Rating Scale^24^). Alongside the development of technology, there has been a search for quick and objective measurement methods^25–27^. However, most of these measurement methods have been used to evaluate ataxia in diseases other than MS^27–29^. The numbers of studies evaluating upper extremity ataxia in patients with MS are limited. Ueda et al. instructed 49 people with spinocerebellar degeneration to follow a spiral pattern. They analyzed the area between the spiral lines by means of the Image J software. These areas were correlated using SARA and cerebellar volume^25^.

Erdeo et al. evaluated upper extremity ataxia using areas of deviation from the spiral line on the tablet and could not find any significant difference in the patients’ dominant hand data using ICARS and EDSS^26^. Our study was an advanced version of the study by Erdeo et al. and had the aim of creating a gold standard test. Nguyen et al. investigated upper extremity ataxia using a sensor mounted on a spoon and then evaluated the patients’ kinematics information using an internet-enabled phone. The data obtained were correlated with the results from the NHPT and the box-block test^27^. In our study, the data obtained through the TAAP on the X and Y axes were found to correlate with data obtained via the NHPT.

In a study conducted by Maurel et al. on 19 patients with Friedreich’s ataxia and 15 healthy people, these individuals were instructed to perform three tasks (circling, marking and forearm supination). The joint angle and extremity speeds while performing these tasks were evaluated^23^. Especially in ataxia patients, both the duration of duty and the number of drawing and marking errors were greater. The clinical application was more difficult than in our study, in which only a tablet was required to evaluate ataxia^30–32^.

There are studies evaluating all parameters with ataxia, as well as studies involving the lower or upper extremities^30–32^. In a study by Krishna et al., finger-nose and knee-heel ataxia tests were performed by connecting a sensor to the hand and ankle of the patient. The results were correlated with the sub-parameter of SARA^33^. In particular, the angular velocity increased and rotational movement in the Y axis was found to be higher than in the X and Z axes.

It has also been reported that ataxic movements are more common in the non-dominant extremities. In our study, the Y-axis parameter was more correlated with the ICARS scale in the dominant and non-dominant hand than was the X axis. The reason for this was that upper extremity ataxia caused more deviations in the Y axis than in the X axis. In our study, the data from the non-dominant hand were found to be higher than those from the dominant hand. In this regard, our study is similar to that of Krishna. We think that this difference was not due to excessive ataxic movements in the non-dominant hand but, rather, to weak motor skills of the hand. In this aspect, our study differs from the literature^23,25^.

Different studies have used video analysis methods, sensors and optoelectronic systems in ataxia evaluations^34–36^. In all those studies, the numbers of individuals included in the study were less than in our study. Additionally, those studies required special instruments and sophisticated analysis methods. This makes it difficult to apply ataxia assessments in a clinical setting. Our method is advantageous in that it is easier to apply. If TAAP is installed on phones as an application, all experts working in the clinic can easily apply it. TAAP can be used in patient evaluations and also, with an additional dashboard, it can be used for rehabilitation purposes among patients, through biofeedback.

This study had some limitations. We evaluated the X and Y axis parameters, but we did not include the Z axis in the evaluation because it is difficult to ensure the reliability of the Z axis in terms of depth perception with a single camera. In addition, just as it is not possible to separate balance and coordination with precise boundaries, pyramidal problems cannot be completely ruled out.

In conclusion, we designed a low-cost system that enabled evaluation of upper extremity ataxia quickly, easily and objectively among patients with ataxic MS. The correlations of the data that we obtained, both with other objective tests and with commonly used clinical scales were promising. We anticipate that TAAP will become the first-choice tool for clinicians and physiotherapists in evaluating and following up diseases that involve ataxia, including both MS and other diseases, as well as in clinical rehabilitation studies.
